# Low Temperature Storage Stimulates Fruit Softening and Sugar Accumulation Without Ethylene and Aroma Volatile Production in Kiwifruit

**DOI:** 10.3389/fpls.2019.00888

**Published:** 2019-07-05

**Authors:** Oscar W. Mitalo, Sumire Tokiwa, Yuki Kondo, Takumi Otsuki, Ivan Galis, Katsuhiko Suezawa, Ikuo Kataoka, Anh T. Doan, Ryohei Nakano, Koichiro Ushijima, Yasutaka Kubo

**Affiliations:** ^1^Graduate School of Environmental and Life Science, Okayama University, Okayama, Japan; ^2^Institute of Plant Science and Resources, Okayama University, Kurashiki, Japan; ^3^Faculty of Agriculture, Kagawa University, Miki, Japan

**Keywords:** ethylene, ethyl butanoate, low temperature, methyl butanoate, softening, sugars

## Abstract

Fruit ripening in response to propylene (an ethylene analog), 1-methylcyclopropene (1-MCP, an ethylene action inhibitor), and low temperature (5°C) treatments was characterized in “Kosui” kiwifruit (*Actinidia rufa* × *A. chinensis*). Propylene treatment induced ethylene production, with increased expression levels of *1-aminocyclopropane-1-carboxylic acid* (*ACC*) *synthase 1* (*AcACS1*) and *ACC oxidase 2* (*AcACO2*), and rapid fruit softening together with increased expression levels of *polygalacturonase* (*AcPG*) and *expansin 1* (*AcEXP1*) within 5 days (d). Fruit soluble solids concentration (SSC) and contents of sucrose, glucose, and fructose together with the expression levels of *β-amylase 1* (*Acβ-AMY1*), *Acβ-AMY2*, and *invertase* (*AcINV3-1*) increased rapidly after 5 d exposure to propylene. Furthermore, propylene exposure for 5 d was sufficient to induce the production of key aroma volatile compounds, ethyl- and methyl butanoate, accompanied with increased expression levels of *alcohol acyl transferase* (*AcAAT*). Application of 1-MCP at the start of the experiment, followed by continuous exposure to propylene, significantly delayed fruit softening, changes in SSC and sugars, and strongly suppressed the production of ethylene, aroma volatiles, and expression of associated genes. During storage, fruit softening, SSC and sugar increase, and increased expression of genes associated with cell wall modification and carbohydrate metabolism were registered without detectable ethylene production; however, these changes occurred faster at 5°C compared to 22°C. Interestingly, ethyl and methyl butanoate as well as *AcAAT* expression were undetectable in kiwifruit during storage, while they were rescued by post-storage propylene exposure, indicating that the production of aroma volatile compounds is strongly ethylene-dependent. Transcript levels of a NAC-related transcription factor (TF), *AcNAC3*, increased in response to both propylene and low temperature treatments, while *AcNAC5* was exclusively up-regulated by propylene. By contrast, transcript levels of a MADS-box TF, *AcMADS2*, exclusively increased in response to low temperature. The above findings indicate that kiwifruit ripening is inducible by either ethylene or low temperature signals. However, fruit ripened by low temperature were deficient in ethylene-dependent aroma volatiles, suggesting that ethylene signaling is non-functional during low temperature-modulated ripening in kiwifruit. These data provide further evidence that ethylene-dependent and low temperature-modulated ripening in kiwifruit involve different regulatory mechanisms.

## Introduction

The plant hormone ethylene regulates a wide range of plant growth and developmental processes, including fleshy fruit ripening (Lashbrook et al., 1998; Giovannoni, 2004). During ripening, fleshy fruit undergo various physiological, biochemical, and structural changes including softening, starch degradation to sugars, pigment accumulation, and production of aroma volatiles (Klee and Giovannoni, 2011; Osorio et al., 2013). The onset of fruit ripening in climacteric fruit such as tomatoes, apples, and peaches is accompanied by a marked increase in ethylene production (Xu et al., 2012), which is regulated at the transcriptional level via the differential expression of genes encoding two key enzymes: 1-aminocyclopropane-1-carboxylic acid (ACC) synthase (ACS) and ACC oxidase (ACO) (Wang et al., 2002; Cherian et al., 2014).

Kiwifruit (*Actinidia* spp.) are categorized as climacteric, since fruit ripening is largely driven by ethylene-regulated changes in gene expression (Antunes, 2007; Yin et al., 2008). Exogenous ethylene or propylene treatment initiates rapid fruit softening through the induction of several cell wall modification-associated genes such as *polygalacturonase* (*AcPG*) and *expansin 1* (*AcEXP1*) (Wang et al., 2000; Atkinson et al., 2011; Mworia et al., 2012). In addition, kiwifruit respond to exogenous ethylene/propylene by increasing their soluble solids concentration (SSC), which is associated with the induction of various starch degradation-related genes such as *β-amylase* (*Acβ-AMY*) (Nardozza et al., 2013; McAtee et al., 2015; Hu et al., 2016). Ripe kiwifruit produce a composite of aroma volatiles, consisting of mainly aldehydes and esters (Marsh et al., 2006). Characteristic kiwifruit esters have been identified as ethyl butanoate and methyl butanoate (Zhang et al., 2009), and their regulation by ethylene has been previously described (Atkinson et al., 2011; Günther et al., 2011, 2015). Ethylene-induced ripening changes in kiwifruit are usually followed by a sharp increase in ethylene production caused by the up-regulation of key biosynthetic genes *AcACS1* and *AcACO2* (Pratt and Reid, 1974; Mworia et al., 2010; Atkinson et al., 2011; McAtee et al., 2015).

Kiwifruit exhibit a peculiar ripening behavior, as substantial softening in healthy intact fruit occurs during low temperature storage in the absence of any detectable ethylene (Antunes, 2007; Yin et al., 2009). Arpaia et al. (1987) demonstrated that kiwifruit are sensitive to extremely low ethylene concentrations (as low as 0.01 μLL^-1^). Consequently, the substantial softening during storage at <1.5°C in air (presumed to contain 0.001 μLL^-1^ ethylene) is believed to be controlled by basal levels of system I ethylene that is present in most fruit and plant tissues (Kim et al., 1999; Pranamornkith et al., 2012; Jabbar and East, 2016). However, to date, there is no direct evidence linking system I ethylene to kiwifruit ripening during low temperature storage, and thus, the regulatory mechanisms associated with this phenomenon remain less well understood.

Over the past few years, this study group has been dedicated to elucidating the molecular mechanisms underpinning low temperature-modulated fruit ripening in kiwifruit. A preliminary study by Mworia et al. (2012) demonstrated that healthy intact “Sanuki Gold” kiwifruit softened; accumulated *AcPG, Pectate lyase* (*AcPL*), and *AcEXP1* mRNAs; and decreased their titratable acidity (TA) during storage at 4°C, but not at 25°C, despite the lack of any detectable increase in ethylene production. These changes were not suppressed by frequent application of 1-methylcyclopropene (1-MCP) to keep fruit insensitive to ethylene, indicating that they occur outside the sphere of ethylene influence. Similar results were reported using different kiwifruit cultivars (Asiche et al., 2017; Mitalo et al., 2018, 2019), confirming that low temperature-modulated ripening is common to all kiwifruit cultivars. Follow-up transcriptomic studies revealed that a distinct set of ripening-associated genes in kiwifruit were uniquely regulated by low temperature, independent of ethylene (Asiche et al., 2018). Despite these findings, low temperature-modulated ripening in kiwifruit remains a poorly understood phenomenon. There is a growing need to conduct more focused studies to examine the similarities and differences in the molecular regulation of ethylene-induced and low temperature-modulated ripening in kiwifruit.

Using gas chromatography–mass spectrometry (GC–MS) to analyze the volatiles and soluble sugar contents of “Kosui” kiwifruit, an interspecific hybrid between *Actinidia rufa* and *A. chinensis*, this study sought to investigate the impact of low temperature (5°C) on sugar and aroma volatile profiles relative to ethylene effect. Changes in sugar composition and aroma volatile production in response to propylene, 1-MCP, and during storage at 5 and 22°C were compared, and their concomitant gene expression patterns are reported. Our results indicate that the production of aroma volatiles is strongly ethylene-dependent and is absent during cold storage, providing evidence that ethylene signaling is non-functional during low temperature-modulated ripening.

## Materials and Methods

### Plant Material and Treatments

“Kosui” kiwifruit grown under standard cultural practices were harvested from a commercial orchard in Takamatsu, Japan at a physiological maturity stage [170 days (d) after full bloom (DAFB), firmness: 73.02 ± 3.39 N, SSC: 7.79 ± 0.18%, TA: 2.20 ± 0.03%]. After harvesting, careful sorting was conducted to exclude fruit with physical injuries, disease symptoms, and those producing ethylene. Fruit were then divided into five sets, corresponding to the various treatments.

#### Ethylene-Dependent Ripening

Three sets of 30 fruit each were used in this experiment. The first set was held in gas-tight plastic containers that were continuously treated with 5000 μLL^-1^ propylene, a well-known ethylene analog (McMurchie et al., 1972; Mworia et al., 2010; Asiche et al., 2016). Propylene treatment was done to induce ethylene signaling, and to allow for determination of endogenous ethylene produced by the fruit. The second set was initially exposed to 2 μLL^-1^ 1-MCP for 12 h followed by continuous exposure to 5000 μLL^-1^ propylene. 1-MCP was released by dissolving SmartFresh^TM^ powder (AgroFresh, PA, United States) in water. The third set contained non-treated fruit as a control. All treatments were carried out at 22°C for up to 9 d. Soda lime was placed in plastic containers during propylene and 1-MCP treatments to reduce CO_2_ accumulation.

#### Low Temperature-Modulated Ripening

Two sets of 300 fruit each were stored either at 5 or 22°C in air for up to 49 d. During storage, fruit were individually wrapped in perforated polythene bags to reduce water loss, before placing them (∼10 cm apart) in plastic trays. Ethylene production pattern of each fruit was monitored at weekly intervals throughout the storage period. To avoid ethylene accumulation in the storage chambers, fruit that produced detectable ethylene (<10%) were excluded based on previous observations that ethylene production correlated with the appearance of disease symptoms (Asiche et al., 2018). At the end of the storage period, fruit at 5°C were further divided into two groups before being transferred to 22°C for up to 14 d; one group was continuously treated with propylene as described above, while the other group was left untreated. Similarly, fruit at 22°C were divided into two groups; one group was continuously treated with propylene and the other one was left untreated.

### Evaluation of Changes in Ethylene Production, Firmness, SSC, and TA

To determine ethylene production, individual fruit were incubated in a 440-mL container for up to 1 h. Ethylene production rate was measured by withdrawing 1 mL of headspace gas and injecting it into a gas chromatograph (Model GC4 CMPF, Shimadzu, Kyoto, Japan), equipped with a flame ionization detector (set at 200°C) and an activated alumina column (set at 80°C) (Mworia et al., 2012). This procedure has a minimum ethylene detection capacity of 0.01 nLg^-1^ h^-1^. Fruit firmness was measured at two equatorial regions of peeled fruit using a penetrometer (model SMT-T-50, Toyo Baldwin, Tokyo, Japan) fitted with an 8-mm plunger. Data were recorded as Newtons (N) and firmness was expressed as a mean of five independent biological replications. SSC and TA were determined using fruit juice as described elsewhere (Asiche et al., 2018; Mitalo et al., 2019).

### Collection of Aroma Volatiles

Aroma volatile compounds were collected according to the procedure by Sobhy et al. (2017), with slight modifications. Intact fruit were placed into sealed 1360-mL containers equipped with two ports; one for the air inlet and another port for the outlet. Dried air, purified by passing through a charcoal filter, was introduced to sweep through the headspace at approximately 0.75 L min^-1^. The air was then pulled out through the outlet port fitted with a custom-made 10-cm glass trap (5 mm inner diameter), filled with Porapak Q (200 mg, Supelco Analytical, Bellefonte, PA, United States) held in place by two plugs of silanized glass wool. Porapak Q traps were conditioned before use by flushing with 2 mL methanol (Sigma–Aldrich Co., United States), followed by 2 mL dichloromethane (DCM, Wako Pure Chemical Industries, Japan), dried, and then placed overnight in an oven at 60°C for complete drying. To exclude the effect of background volatiles in air, empty containers having no fruit were included in the collection setup. Aroma volatiles were collected over 24 h periods. After each collection period, volatile compounds were eluted from Porapak Q traps with 1 mL DCM after adding 400 ng tetralin (1,2,3,4-tetrahydronaphthalene; Nacalai Tesque, Japan) on the top of each column. Tetralin was used as an internal standard. The samples were stored in 1.5 mL glass vials at -20°C until further analysis.

### Extraction and Derivatization of Soluble Sugars

Soluble sugars were obtained and derivatized as described by Wang et al. (2010). Briefly, the samples (0.1 g) were ground in liquid nitrogen and extracted in 1.4 mL of 100% methanol with ribitol (12 μg) added as an internal standard. After fractionating the non-polar metabolites into chloroform, 150 μL of the polar phase was transferred into a 1.5 mL micro-centrifuge tube to measure the metabolites (soluble sugars). These were dried under vacuum without heating, flushed with nitrogen gas, and then derivatized sequentially with methoxyamine hydrochloride and *N*-methyl-*N*-trimethylsilyl-trifluoroacetamide (Lisec et al., 2006). The samples were stored at -20°C until further analysis.

### GC–MS Conditions and Chemical Analysis

Volatile eluates and metabolites were analyzed using an Agilent 240 GC–MS ion trap system coupled with Agilent 7891A GC fitted with the HP-5MS column (5% phenyl methyl silox, 30 m length × 0.25 mm inner diameter × 0.25 μm film thickness) (Agilent Technologies, Santa Clara, CA, United States). One microliter of each eluted sample was injected in split mode (1:30) into the injector port of the GC instrument held at 230°C via an Agilent 7693A auto-sampler. Helium (1 mL/min) was used as carrier gas, and MS ionization was achieved by electron impact (EI) at emission current 30 μAmps for volatiles (10 μAmps for metabolites) in the ion trap held at 200°C (transfer line was 260°C). For headspace volatile analyses, the GC oven temperature was programmed at 40°C for 3 min, and then increased at 5°C min^-1^ to 180°C, followed by 20°C min^-1^ ramp to 300°C, where it was held for an additional 5 min before returning to initial conditions. For metabolite samples, GC temperature program was 5 min at 60°C, followed by 5°C min^-1^ to 300°C, 5 min hold period, and return to initial temperature and equilibration. MS data were collected in full scan mode in mass range *m*/*z* 40–300 for volatiles (*m*/*z* 40–750 for metabolites), and analyzed by Agilent Workstation Version 7.0.2 software. Aroma volatiles and metabolites were identified by comparing their fragmentation patterns with those from the NIST 2011 Mass Spectral Library and Software (National Institute of Standards and Technology, United States). Co-injection with authentic standards was undertaken to confirm tentative identifications where possible. Quantifications were based on standard curves generated for each target compound and internal standards.

### RNA Extraction

Total RNA was extracted from ∼3 g of outer pericarp tissue (three biological replications) using a method for polysaccharide-rich tissues (Ikoma et al., 1996), with slight modifications. DNase I (Nippon Gene, Tokyo) treatment followed by clean-up using FavorPrep after Tri-Reagent RNA Clean-up Kit (Favorgen Biotech Co., Pingtung, Taiwan) were carried out to remove genomic DNA contamination from the extracted RNA.

### Quantitative Real-Time PCR (RT-qPCR)

The method used was similar to that reported in our previous study (Asiche et al., 2018). Briefly, first strand cDNA was synthesized from 2.4 μg of RNA using RevTra Ace reverse transcriptase (Toyobo, Osaka, Japan), and random hexamer primer according to the manufacturer’s instructions. Gene-specific primers (Supplementary Table 1) for RT-qPCR analysis were designed using Primer3 software (version 0.4.0^1^). Gene expression of three biological replications was examined on a MyiQ Single-Color Reverse Transcriptase-Quantitative PCR Detection System (Bio-Rad, Hercules, CA, United States) using TB Green^TM^ Premix Ex Taq^TM^ II (Tli RNaseH Plus) (TaKaRa, Shiga, Japan) according to the manufacturer’s instructions. *AcActin* was used as the housekeeping gene. The specificity of all primers was verified by melting curve analysis. Relative gene expression was calculated using the 2^-ΔΔCt^ method with samples at harvest (0 d) calibrated as 1.

### Statistical Analysis

Data obtained in this study were subjected to statistical analyses using R software (version 3.4.0, R Project). ANOVA followed by *post hoc* Tukey’s tests (*p* ≤ 0.05) were used to detect differences in fruit ripening characteristics and gene expression levels among the different treatments.

## Results

### Induction of Ethylene Biosynthesis in Postharvest Kiwifruit

**FIGURE 1:**
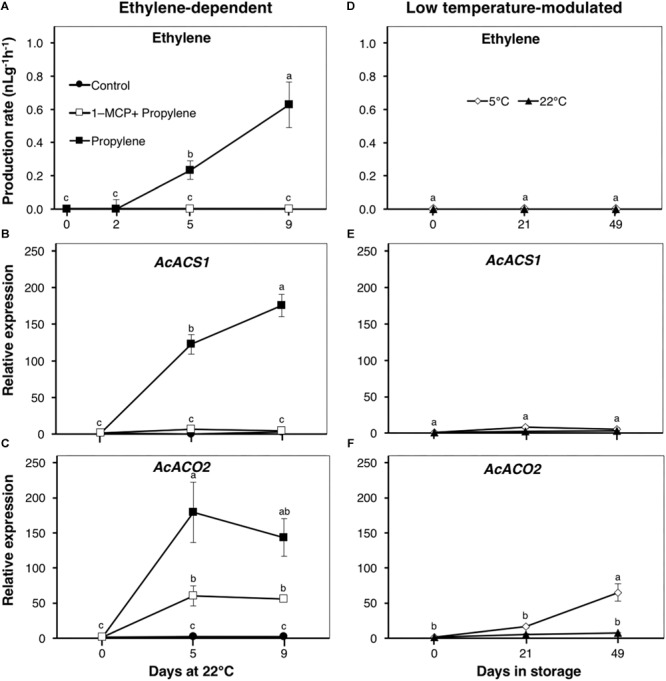
Ethylene production and expression patterns of ethylene biosynthetic genes in kiwifruit. **(A)** Ethylene production pattern during ethylene-dependent ripening. Control: non-treated; propylene: continuously treated with propylene (5000 μLL^-1^, 22°C) for up to 9 d; 1-MCP+Propylene: a single exposure to 1-methylcyclopropene (1-MCP, 2 μLL^-1^) for 12 h immediately after harvest, followed by continuous propylene treatment for up to 9 d. **(D)** Ethylene production during low temperature-modulated ripening. Kiwifruit immediately after harvest were separately stored at either 5 or 22°C in ethylene-free air for up to 49 d. Relative transcript levels of ethylene biosynthetic genes, *1-aminocyclopropane-1-carboxylate* (*ACC*) *synthase 1* (*AcACS1*, Achn364251) and *ACC oxidase 2* (*AcACO2*, Achn326461), were determined against at-harvest (0 d) samples by RT-qPCR using kiwifruit *actin* (EF063572) as an endogenous control **(B,C,E,F)**. Data are mean (±SE) of at least three independent biological replications. Error bars not shown are smaller than the symbol used. Different letters indicate significant differences in ANOVA (Tukey’s test, *p* < 0.05).

At harvest, kiwifruit used in this study did not produce any detectable ethylene levels. To understand the molecular mechanisms responsible for ethylene production during postharvest handling, we determined the effects of propylene and 1-MCP treatments, as well as storage temperature (Figure 1).

As expected, endogenous climacteric ethylene production was detected in propylene-treated fruit at a level of 0.23 nLg^-1^ h^-1^ after 5 d, increasing to 0.63 nLg^-1^ h^-1^ after 9 d (Figure 1A). No endogenous ethylene production was measured in fruit pre-treated with 1-MCP, nor in the control fruit, throughout the experimental period. The expression of ethylene biosynthetic genes, *AcACS1* and *AcACO2*, significantly increased in propylene-treated fruit after 5 and 9 d by 123–176- and 143–179-fold, respectively (Figure 1B,C). *AcACS1* expression showed no measurable changes in fruit pre-treated with 1-MCP, while *AcACO2* expression was significantly reduced to <60-fold. During storage, no endogenous ethylene production was measured in healthy intact fruit either at 5 or 22°C throughout the experimental period (Figure 1D). There were no transcriptional changes observed in *AcACS1* (Figure 1E), while *AcACO2* showed a considerable expression increase (65-fold) in fruit at 5°C after 49 d (Figure 1F). These observations indicate that production of climacteric ethylene in kiwifruit requires ethylene signaling (triggered by propylene), and is largely dependent upon the transcriptional regulation of *AcACS1*.

### Kiwifruit Softening Is Inducible by Either Ethylene or Low Temperature

**FIGURE 2:**
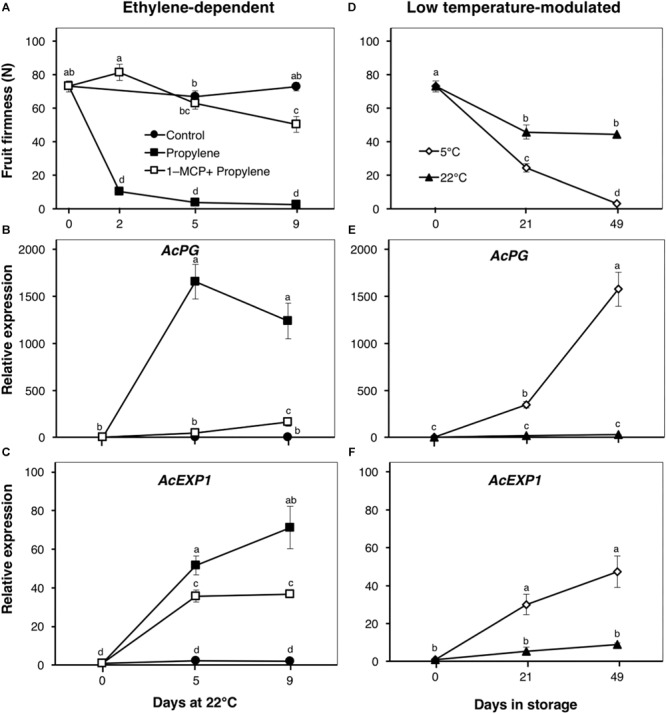
Fruit softening and expression patterns of genes encoding cell wall modifying enzymes in kiwifruit. **(A)** Ethylene-dependent softening in kiwifruit. Control: non-treated; propylene: continuously treated with propylene (5000 μLL^-1^, 22°C) for up to 9 d; 1-MCP+Propylene: a single exposure to 1-MCP (2 μLL^-1^) for 12 h immediately after harvest, followed by continuous propylene treatment for up to 9 d. **(D)** Low temperature-modulated softening in kiwifruit. Kiwifruit immediately after harvest were separately stored at either 5 or 22°C in ethylene-free air for up to 49 d. Relative transcript levels of kiwifruit *polygalacturonase* (*AcPG*, Achn051381) and *expansin 1* (*AcEXP1*, Achn336951) were determined against at-harvest (0 d) samples by RT-qPCR using kiwifruit *actin* (EF063572) as an endogenous control **(B,C,E,F)**. Data are mean (±SE) of at least three independent biological replications. Error bars not shown are smaller than the symbol used. Different letters indicate significant differences in ANOVA (Tukey’s test, *p* < 0.05).

Following propylene treatment, kiwifruit firmness rapidly decreased from 73 to 10 N within 2 d, and to <3 N after 5 d (Figure 2A). Fruit pre-treated with 1-MCP showed only a slight decrease in firmness, to 50 N after 9 d. The cell wall modification-associated genes, *AcPG* and *AcEXP1*, showed a marked increase in expression (>1000- and 71-fold, respectively) during propylene treatment after 5 and 9 d (Figure 2B,C). The induction of both genes by propylene was significantly reduced by 1-MCP pre-treatment. Control fruit showed no significant changes in firmness, and the expression of both *AcPG* and *AcEXP1* was maintained at minimal levels throughout the experimental period.

The firmness of fruit stored at 5°C showed a substantial decrease from 73 to 24 N after 21 d, and to 3 N after 49 d (Figure 2D). At 22°C, fruit firmness showed only a slight decrease to 45 N after 21 d with no further changes thereafter. Both *AcPG* and *AcEXP1* expression showed sustained increases in fruit at 5°C, whereas their expression in fruit at 22°C were maintained at low levels throughout the storage period (Figure 2E,F).

### Changes in the Composition of Major Soluble Sugars

**FIGURE 3:**
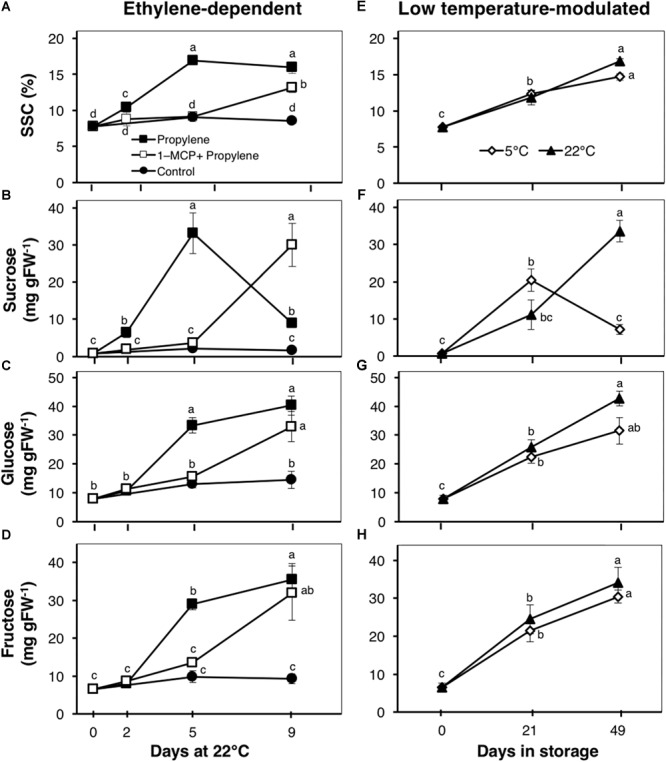
Total soluble solids concentration (SSC) and composition of major sugars in kiwifruit. **(A–D)** Changes in SSC and concentration of sucrose, glucose, and fructose during ethylene-dependent ripening. Control: non-treated; propylene: continuously treated with propylene (5000 μLL^-1^, 22°C) for up to 9 d; 1-MCP+Propylene: a single exposure to 1-MCP (2 μLL^-1^) for 12 h immediately after harvest, followed by continuous propylene treatment for up to 9 d. **(E–H)** Changes in SSC and concentration of sucrose, glucose, and fructose during low temperature-modulated ripening. Kiwifruit immediately after harvest were stored at either 5 or 22°C. Data are mean (±SE) of five independent biological replications. Error bars not shown are smaller than the symbol used. Different letters indicate significant differences in ANOVA (Tukey’s test, *p* < 0.05).

The SSC of propylene-treated fruit increased rapidly from 7.8% to a maximum 16.5% after 5 d (Figure 3A). This increase was significantly delayed by 1-MCP pre-treatment, with fruit showing little change for the first 5 d; a substantial increase to 13.2% was observed after 9 d. There was a rapid increase in sucrose content of propylene-treated fruit, from 0.7 to 33.1 mg gFW^-1^ after 5 d, also followed by a sudden decrease to 8.9 mg gFW^-1^ after 9 d (Figure 3B). Both glucose and fructose contents showed a sustained increase in propylene-treated fruit to 40 and 36 mg gFW^-1^, respectively, after 9 d (Figure 3C,D). The increase in sucrose, glucose, and fructose contents by propylene treatment was significantly delayed by 1-MCP pre-treatment, although the fruit eventually attained almost similar contents at the end of the experimental period. No measurable changes in SSC, as well as the contents of sucrose, glucose, and fructose, were observed in non-treated fruit. During storage, SSC and the contents of glucose and fructose exhibited a sustained increase with no significant differences between fruit at 5 and 22°C (Figure 3E,G,H), while the sucrose content rose sharply in fruit at 5°C to a maximum 20.4 mg gFW^-1^ after 21 d followed by a decrease to 7.2 mg gFW^-1^ after 49 d (Figure 3F). The sucrose content steadily increased in fruit at 22°C to 33.5 mg gFW^-1^ after 49 d.

**FIGURE 4:**
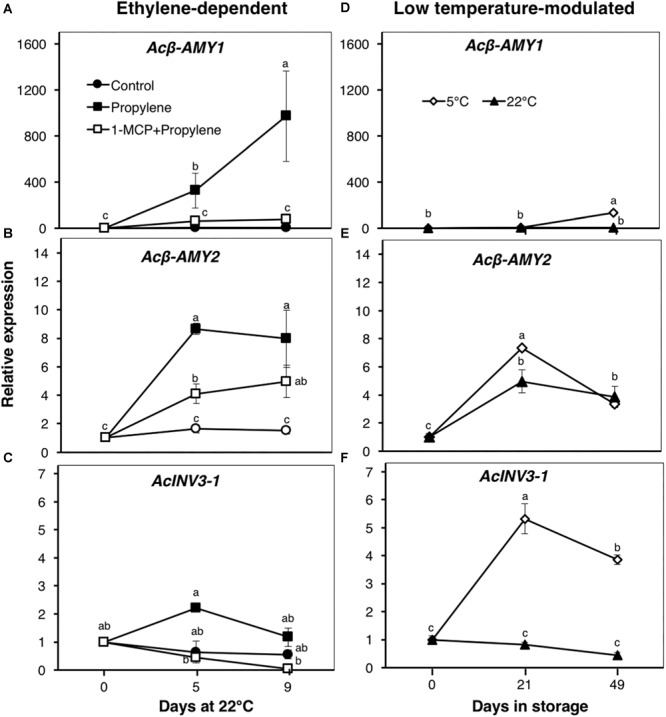
Expression patterns of genes encoding starch degradation and sucrose metabolism enzymes in kiwifruit. Relative transcript levels of *β-amylase 1* (*Acβ-AMY1*, Achn141771 and *Acβ-AMY2*, Achn212571) **(A,B,D,E)** and *invertase 3-1* (*AcINV3-1*, Achn319711) **(C,F)** were determined against at-harvest (0 d) samples by RT-qPCR using kiwifruit *actin* (EF063572) as an endogenous control. Data are mean (±SE) of three independent biological replications. Error bars not shown are smaller than the symbol used. Different letters indicate significant differences in ANOVA (Tukey’s test, *p* < 0.05).

Since the content of soluble sugars was significantly affected by propylene and storage, we examined the expression patterns of genes associated with starch degradation (*Acβ-AMY1* and *Acβ-AMY2*) and sucrose metabolism (*AcINV3-1*) (Figure 4). *Acβ-AMY1* and *Acβ-AMY2* both showed an increase in expression in propylene-treated fruit as the soluble sugars increased, while they were significantly suppressed in fruit pre-treated with 1-MCP (Figure 4A,B). During storage, there was a small increase in *Acβ-AMY1* expression in fruit at 5°C after 49 d, whereas no significant expression changes were observed in fruit at 22°C (Figure 4D). On the other hand, *Acβ-AMY2* expression increased in fruit at both 5 and 22°C, to a maximum (five to sevenfold) after 21 d, and later decreased after 49 d (Figure 4E). *AcINV3-1* showed only a small expression increase (twofold) in propylene-treated fruit after 5 d, while no significant changes were observed in fruit pre-treated with 1-MCP (Figure 4C). However, *AcINV3-1* expression significantly increased (by fivefold after 21 d, and fourfold after 49 d) during storage of fruit at 5°C; no measurable changes in expression were recorded in fruit at 22°C (Figure 4F).

### Aroma Volatile Production Is Strongly Ethylene-Dependent, and Is Undetectable During Low Temperature-Modulated Fruit Ripening

**FIGURE 5:**
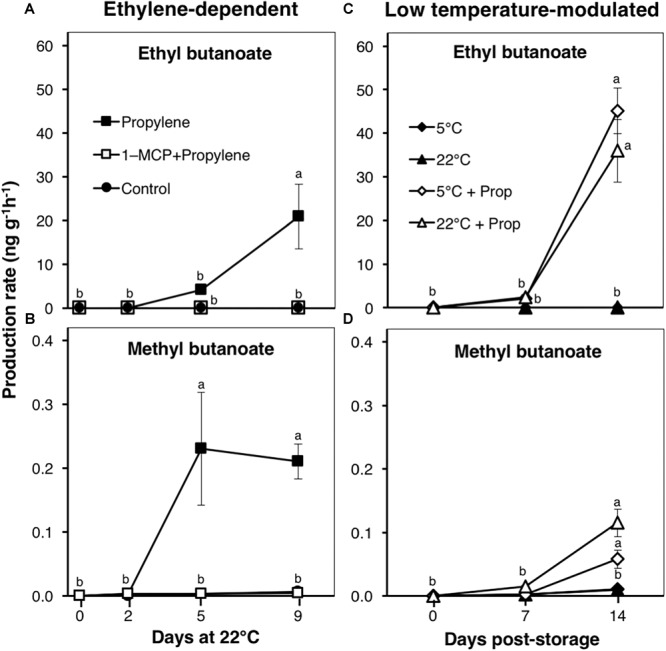
Aroma volatile production patterns in kiwifruit. **(A,B)** Changes in production rates of ethyl- and methyl butanoate during ethylene-dependent ripening. Control: non-treated; propylene: continuously treated with propylene (5000 μLL^-1^, 22°C) for up to 9 d; 1-MCP+Propylene: a single exposure to 1-MCP (μLL^-1^) for 12 h immediately after harvest, followed by continuous propylene treatment for up to 9 d. **(C,D)** Ethyl- and methyl butanoate production rates in kiwifruit after storage at either 5 or 22°C. After 49 d storage, fruit were transferred to 22°C with or without propylene-treatment. Data are mean (±SE) of three independent biological replications containing at least three fruit each. Error bars not shown are smaller than the symbol used. Different letters indicate significant differences in ANOVA (Tukey’s test, *p* < 0.05).

Fruit aroma volatiles were identified and quantified by GC–MS during ethylene-dependent ripening, after 49 d storage at either 5 or 22°C, and during 14 d shelf life (at 22°C) (Figure 5). The major volatiles detected were esters; particularly ethyl butanoate and methyl butanoate (Supplementary Figures 1, 2), which are considered to form part of the characteristic ripe fruit flavor for kiwifruit (Zhang et al., 2009; Atkinson et al., 2011). Propylene-treated fruit produced large amounts of ethyl butanoate at a rate of 4.2 ngg^-1^ h^-1^ after 5 d, and 20.9 ngg^-1^ h^-1^ after 9 d (Figure 5A). Similarly, methyl butanoate was detected at a rate of 0.2 ngg^-1^ h^-1^ in propylene-treated fruit after 5 d; no further increase was observed thereafter (Figure 5B). No measurable increase in the production of both esters was observed in fruit pre-treated with 1-MCP, as well as in control fruit. During storage, ethyl butanoate and methyl butanoate levels were undetectable in fruit at either 5 or 22°C, while they only increased during post-storage treatment with propylene (Figure 5C,D), consistent with endogenous ethylene production (Supplementary Figure 3).

**FIGURE 6:**
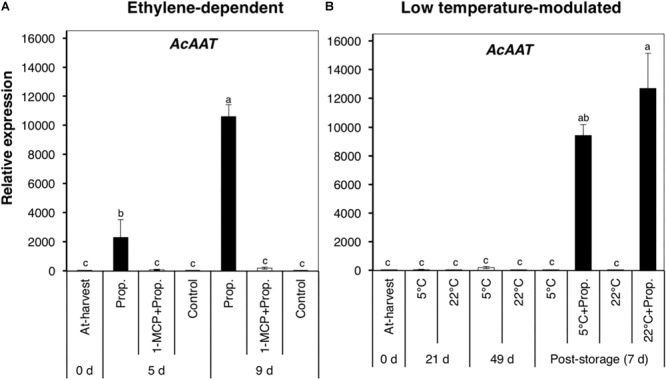
Expression pattern of an alcohol acyl transferase-encoding gene in kiwifruit during ethylene-dependent **(A)** and low temperature-modulated ripening **(B)**. Relative transcript levels of *AcAAT* (KJ626345) were determined against at-harvest (0 d) samples by RT-qPCR using kiwifruit *actin* (EF063572) as an endogenous control. Data are mean (±SE) of three independent biological replications. Error bars not shown are smaller than the symbol used. Different letters indicate significant differences in ANOVA (Tukey’s test, *p* < 0.05).

We also examined the expression of *AcAAT*, which encodes an alcohol acyl transferase associated with ester production during fruit ripening (Souleyre et al., 2005; Günther et al., 2011). *AcAAT* showed a dramatic increase in expression in propylene-treated fruit after 5 d (∼2000-fold) and 9 d (∼11,000-fold), while no significant changes were observed in fruit pre-treated with 1-MCP (Figure 6A). By contrast, *AcAAT* showed no significant changes in expression during storage at either 5 or 22°C, whereas it was massively up-regulated (>10,000-fold) 7 d after post-storage treatment with propylene (Figure 6B).

### Expression Analysis of Fruit Ripening-Associated Transcription Factors

**FIGURE 7:**
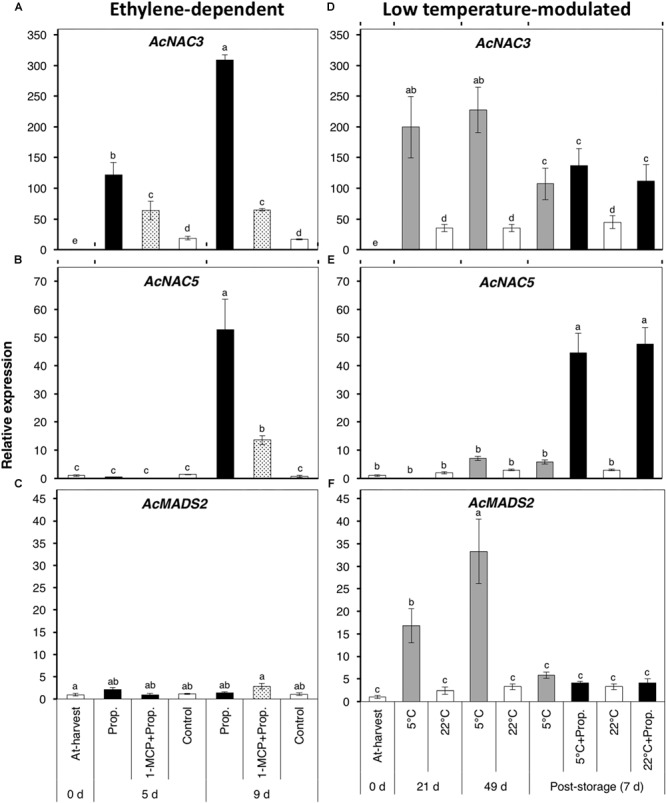
Expression patterns of genes encoding ripening-associated transcription factors (TFs) in kiwifruit during ethylene-dependent and low temperature-modulated ripening. Relative transcript levels of *AcNAC3* (Achn134171) **(A,D)**, *AcNAC5* (Achn169421) **(B,E)**, and *AcMADS2* (Achn235371) **(C,F)** were determined against at-harvest (0 d) samples by RT-qPCR using kiwifruit *actin* (EF063572) as an endogenous control. Data are mean (±SE) of three independent biological replications. Error bars not shown are smaller than the symbol used. Different letters indicate significant differences in ANOVA (Tukey’s test, *p* < 0.05).

As various TFs including MADS-box and NAC domains have been shown to be key regulators of fruit ripening in kiwifruit and other fruit species (Fujisawa et al., 2013; McAtee et al., 2015; Nieuwenhuizen et al., 2015), we determined the expression of three TF-encoding genes (Figure 7). *AcNAC3* was considerably up-regulated (121- and 309-fold after 5 and 9 d, respectively) in propylene-treated fruit (Figure 7A). Its expression was significantly suppressed (64-fold) in fruit pre-treated with 1-MCP, while only a small increase (18-fold) was observed in the control fruit. During storage, *AcNAC3* was also remarkably up-regulated (>200-fold) in fruit at 5°C both after 21 and 49 d, as well as after 7 d post-storage propylene treatment; only a small expression increase (<40-fold) was observed in fruit at 22°C (Figure 7D). A second NAC-related gene, *AcNAC5*, showed an expression increase (53-fold) 9 d after propylene-treatment, while it was significantly inhibited (14-fold) by 1-MCP pre-treatment (Figure 7B). During storage, *AcNAC5* showed no significant change in expression in fruit at either 5 or 22°C, except in post-storage propylene-treated fruit where it was up-regulated (45-fold) after 7 d (Figure 7E). *AcMADS2* showed a completely different expression pattern, as it showed no specific response to propylene treatment (Figure 7C), while it was up-regulated in fruit at 5°C after 21 d (17-fold) and 49 d (33-fold) (Figure 7F). There was no significant change in expression of *AcMADS2* in fruit stored at 22°C. It is also worth noting that *AcMADS2* expression in fruit at 5°C substantially dropped after transfer of the fruit to 22°C.

## Discussion

The ripening behavior of kiwifruit has elicited great interest, as substantial fruit softening during cold storage essentially occurs in the absence of any detectable ethylene; exogenous or endogenous (Antunes, 2007; Yin et al., 2009). In the present work, our results using “Kosui” kiwifruit are consistent with previous research; fruit softening and the expression of cell wall modification-associated genes were induced during storage at 5°C (Figure 2), despite the lack of any measurable increase in ethylene production (Figure 1). It has been suggested that fruit softening during low temperature storage is brought about by basal levels of system I ethylene (Kim et al., 1999), based on the high sensitivity of kiwifruit to ethylene (Pranamornkith et al., 2012; Jabbar and East, 2016). However, the present work strongly suggests that ethylene signaling is non-functional during low temperature-modulated ripening in kiwifruit.

In climacteric fruit, the ethylene biosynthetic pathway is well known to be subject to positive feedback regulation (Kende, 1993). In tomato (Barry et al., 2000), pear (Hiwasa et al., 2003), and banana (Inaba et al., 2007), exposure of fruit to exogenous ethylene/propylene triggers a sharp increase in ethylene production with increased expression of ethylene biosynthetic genes. In kiwifruit, the up-regulation of ethylene biosynthetic genes, *AcACS1* and *AcACO2*, by propylene exposure, coupled with their significant inhibition by 1-MCP (Figure 1B,C), strongly indicate that ethylene biosynthesis is under the influence of ethylene signaling. These observations agree with previous reports suggesting that ethylene biosynthesis during kiwifruit ripening is regulated by a positive feedback mechanism (Whittaker et al., 1997; Mworia et al., 2010). The expression pattern of *AcACS1* suggests that it is strongly ethylene-dependent, as 1-MCP pre-treatment absolutely inhibited its induction by propylene (Figure 1B). Of great importance is the fact that there was no measurable increase in *AcACS1* expression throughout storage of fruit at either 5 or 22°C (Figure 1E), which would account for the undetectable ethylene production (Figure 1D). Therefore, lack of ethylene-dependent *AcACS1* expression, together with undetectable ethylene production during kiwifruit storage, strongly advocate for the idea that ethylene signaling is non-existent. The increase in *AcACO2* expression after 49 d at 5°C (Figure 1F) is insignificant, since it has been shown using tomato that ACS regulates the rate-limiting step in ethylene biosynthesis (Yip et al., 1992; Wang et al., 2002).

It has been shown in previous studies that during fruit ripening, the production of aroma volatiles (especially esters) is regulated by the ethylene signaling pathway. Aroma volatile production was strongly inhibited in ethylene-suppressed kiwifruit (Atkinson et al., 2011), melon (Pech et al., 2008), and apple (Defilippi et al., 2004; Schaffer et al., 2007) lines. Additionally, transgenic lines treated with exogenous ethylene produced increasing concentrations of aroma volatile compounds (Schaffer et al., 2007; Atkinson et al., 2011). Another study by Defilippi et al. (2005) further demonstrated that the expression of *MdAAT*, together with the activity of the associated enzyme, is a rate-limiting step in ester biosynthesis in apple fruit, and both are regulated by ethylene. In the present work, propylene treatment induced the expression of *AcAAT*, together with the production of ethyl butanoate and methyl butanoate in kiwifruit (Figure 5, 6), and their complete suppression by 1-MCP confirmed that they are strongly regulated by the ethylene pathway. It is interesting that the production of these aroma volatiles, as well as the induction of *AcAAT* expression, was not observed in fruit during storage at either 5 or 22°C, further arguing for the lack of ethylene signaling during low temperature-modulated ripening.

Fruit SSC and the concentrations of sucrose, glucose, and fructose increased in response to propylene, as well as during storage at both 5 and 22°C (Figure 3). These changes coincided with increased expression of *Acβ-AMY1* and *Acβ-AMY2* (Figure 4A,B,D,E), which have been previously linked to starch degradation and sugar accumulation in kiwifruit (Nardozza et al., 2013; McAtee et al., 2015; Hu et al., 2016). The above observations suggest that changes in soluble sugars might involve regulatory mechanisms that are independent of both ethylene and low temperature. This is consistent with previous research using different kiwifruit cultivars (Arpaia et al., 1987; Boquete et al., 2004; Mworia et al., 2012; Asiche et al., 2018). However, *Acβ-AMY1* appears to have a stronger response to ethylene since its expression during storage is relatively low (Figure 4A,D). Previous studies in tomatoes (Gao et al., 2007), melons (Pech et al., 2008), and apples (Defilippi et al., 2004) have also demonstrated that there is an ethylene-independent component in starch metabolism and sugar accumulation during fruit ripening. Nevertheless, the expression pattern of *AcINV3-1* suggests that it is more aligned to low temperature response than to ethylene, since its expression increased markedly in fruit at 5°C, but only slightly in response to propylene (Figure 4C,F).

The distinction between ethylene-induced and low temperature-modulated ripening has been difficult to accomplish due to the existence of genes that respond to both stimuli, such as *AcPG* and *AcEXP1* (Figure 2). The expression of *AcNAC3* was also up-regulated by both propylene and low temperature (Figure 7A,D), suggesting its potential role in the regulation of both ethylene-induced and low temperature-modulated ripening. However, its induction during low temperature storage is likely to be independent of ethylene, since it was previously shown that inhibiting ethylene signaling by 1-MCP failed to suppress its upregulation at 5°C (Asiche et al., 2018). NAC-related TFs are involved in regulation of several ripening-associated genes. In tomato, SlNAC4 was shown to regulate fruit ripening and carotenoid accumulation (Zhu et al., 2014), while in banana, several NAC TFs are induced during fruit ripening and are known to physically interact with ethylene insensitive 3 (EIN3)-like (EIL), a major component in the ethylene signaling pathway (Shan et al., 2012). In the present study, the observation that *AcNAC5* was exclusively induced by propylene and not by low temperature (Figure 7B,E) challenges the notion that ethylene signaling is functional during low temperature-modulated fruit ripening. *AcNAC5* expression correlates well with aroma volatile production patterns, suggesting its potential role in regulation of *AcAAT* and aroma volatile biosynthesis during ethylene-dependent ripening in kiwifruit. By contrast, *AcMADS2* expression was substantially induced at 5°C whereas propylene treatment failed to affect its transcript levels (Figure 7C,F), suggesting its potential role in regulation of fruit ripening during cold storage. Our previous studies have also demonstrated the exclusive induction of *AcMADS2* by low temperature in different kiwifruit cultivars (Mitalo et al., 2018, 2019), confirming its alignment to regulatory mechanisms associated with low temperature response. However, since aroma volatiles were undetectable in fruit ripened by low temperature (Figure 5C,D), it appears that *AcMADS2* does not have the capacity to bind or activate *AcAAT* or other aroma-related genes which essentially belong to the ethylene pathway.

**FIGURE 8:**
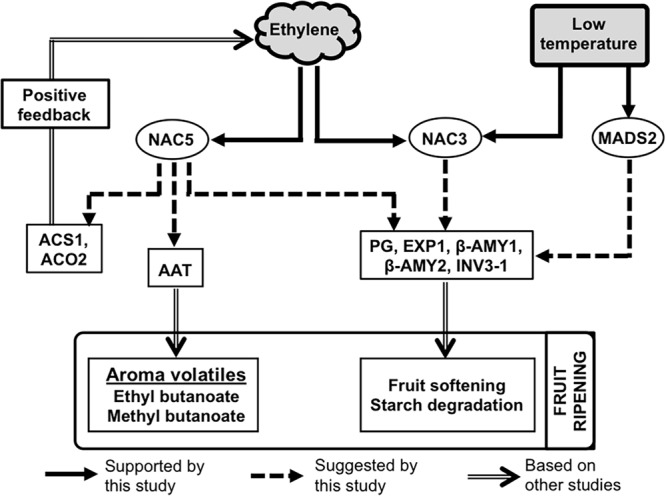
A possible molecular model for the distinct roles of ethylene and low temperature in fruit ripening regulation in kiwifruit. Ethylene signaling recruits a set of specific TFs such as NAC5, as well as shared TFs such as NAC3 which possibly regulate the expression of several genes related with aroma volatile production (*AcAAT*), fruit softening (*AcPG* and *AcEXP1*), and starch degradation (*Acβ-AMY1, Acβ-AMY2, AcINV3-1*). Similarly, a low temperature signal recruits specific TFs including MADS2 and shared TFs such as NAC3 that regulate several ripening-related genes, except those associated with aroma volatile emission.

In summary, the present work has demonstrated that kiwifruit ripening is inducible independently by either ethylene or low temperature signals (Figure 8). Fruit ripened by either stimulus can attain similar quality characteristics in terms of firmness and soluble sugar levels. However, production of aroma volatiles (especially esters: ethyl butanoate and methyl butanoate) and the expression of *AcAAT* appear to be strongly dependent on the ethylene signal. These ethylene-dependent components show negligible changes during low temperature-modulated fruit ripening, providing evidence for the absence of ethylene signaling during cold storage. A distinct group of TFs such as those encoded by *AcNAC5* are exclusively induced by ethylene, suggesting their involvement in regulating ethylene-induced ripening, while a second group encoded by genes such as *AcMADS2* are exclusively aligned to low temperature response. Therefore, it appears that ethylene-induced and low temperature-modulated ripening in kiwifruit involve distinct regulatory mechanisms.

## Data Availability

All datasets (generated/analyzed) for this study are included in the manuscript and/or the Supplementary Files.

## Author Contributions

OM, ST, and YKu conceived and designed the study. OM, ST, YKo, TO, and AD performed most of the experiments with close supervision from YKu, IG, RN, and KU. IG did the GC–MS analysis. KS and IK provided technical assistance. OM wrote the first draft of the manuscript. YKu and IG substantially improved the first draft of the manuscript. All the authors have read and approved the submitted version.

## Conflict of Interest Statement

The authors declare that the research was conducted in the absence of any commercial or financial relationships that could be construed as a potential conflict of interest.

## Footnotes

1 http://bioinfo.ut.ee/primer3-0.4.0/
