# Supplementary material for: Low Temperature Storage Stimulates Fruit Softening and Sugar Accumulation Without Ethylene and Aroma Volatile Production in Kiwifruit
Source: Front Plant Sci. 2019 Jul 5;10:888. doi: 10.3389/fpls.2019.00888 (PMC6625211; doi:10.3389/fpls.2019.00888)
Supplement: Supplementary file 1 [file Data_Sheet_1.ZIP › Supplementary material/Supplementary Figure 3.pptx]

## Slide 1
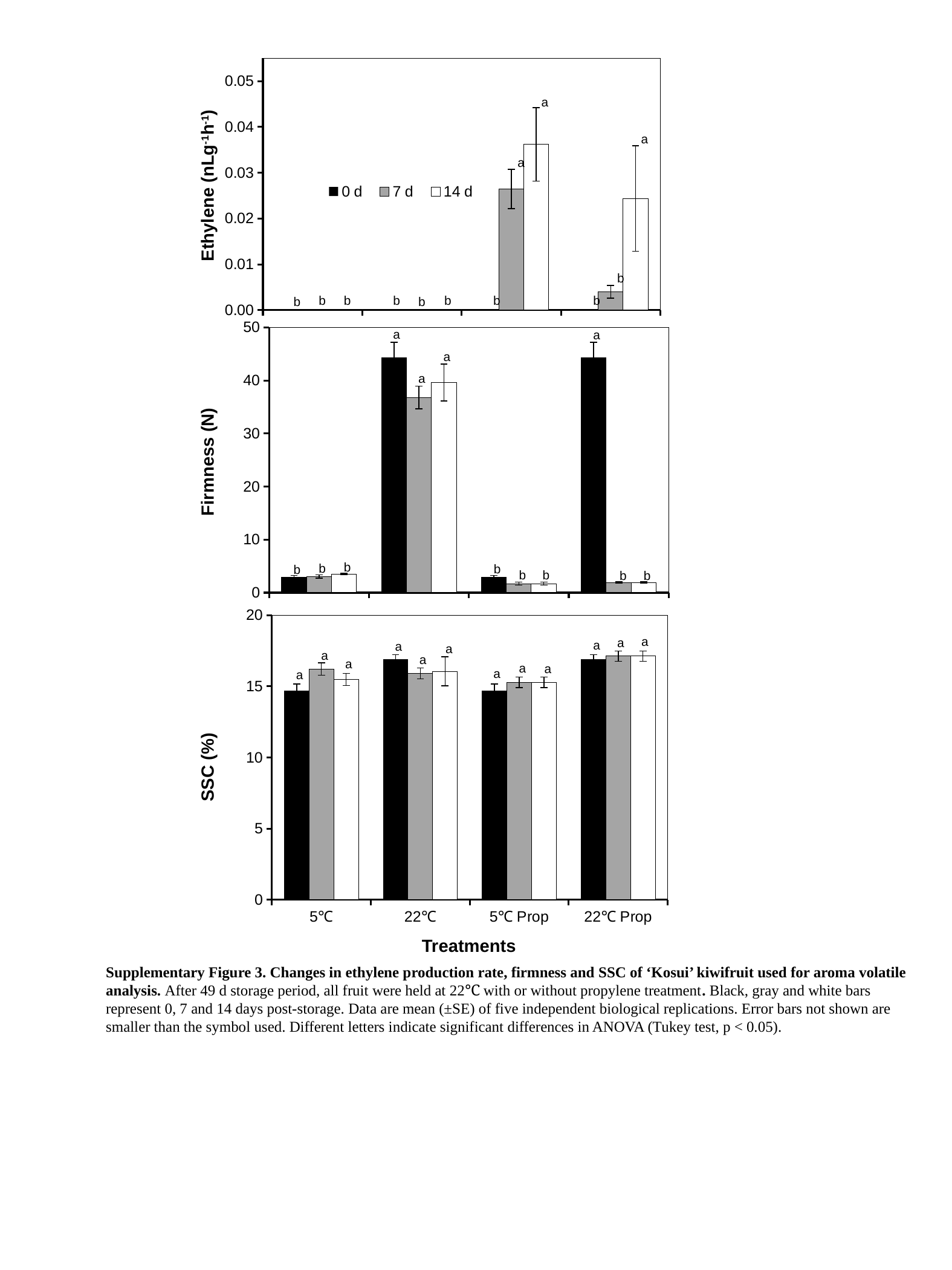

### Chart
| Category | 0 d | 7 d | 14 d |
|---|---|---|---|
| 5℃ | 0.0 | 0.0 | 0.0 |
| 22℃ | 0.0 | 0.0 | 0.0 |
| 5℃ Prop | 0.0 | 0.0264465183163874 | 0.0361558313267552 |
| 22℃ Prop | 0.0 | 0.00399331391677482 | 0.0243652200210436 |Ethylene (nLg-1h-1)
### Chart
| Category | 0 d | 7 d | 14 d |
|---|---|---|---|
| 5℃ | 2.941430656934307 | 3.032992700729928 | 3.519416058394161 |
| 22℃ | 44.35036496350344 | 36.79649635036496 | 39.62919708029197 |
| 5℃ Prop | 2.941430656934307 | 1.688175182481752 | 1.688175182481752 |
| 22℃ Prop | 44.35036496350344 | 1.877021897810219 | 1.877021897810219 |Firmness (N)
### Chart
| Category | 0 d | 7 d | 14 d |
|---|---|---|---|
| 5℃ | 14.69333333333333 | 16.21333333333316 | 15.48666666666667 |
| 22℃ | 16.88666666666667 | 15.90666666666667 | 16.05333333333316 |
| 5℃ Prop | 14.69333333333333 | 15.28666666666667 | 15.28666666666667 |
| 22℃ Prop | 16.88666666666667 | 17.12 | 17.12 |SSC (%)
Treatments
Supplementary Figure 3. Changes in ethylene production rate, firmness and SSC of ‘Kosui’ kiwifruit used for aroma volatile analysis. After 49 d storage period, all fruit were held at 22℃ with or without propylene treatment. Black, gray and white bars represent 0, 7 and 14 days post-storage. Data are mean (±SE) of five independent biological replications. Error bars not shown are smaller than the symbol used. Different letters indicate significant differences in ANOVA (Tukey test, p < 0.05).
a
a
a
b
b
b
b
b
b
b
b
b
a
a
a
a
b
b
b
b
b
b
b
b
a
a
a
a
a
a
a
a
a
a
a
a
