# Supplementary material for: Low Temperature Storage Stimulates Fruit Softening and Sugar Accumulation Without Ethylene and Aroma Volatile Production in Kiwifruit
Source: Front Plant Sci. 2019 Jul 5;10:888. doi: 10.3389/fpls.2019.00888 (PMC6625211; doi:10.3389/fpls.2019.00888)
Supplement: Supplementary file 1 [file Data_Sheet_1.ZIP › Supplementary material/Supplementary Figure 1.pptx]

## Slide 1
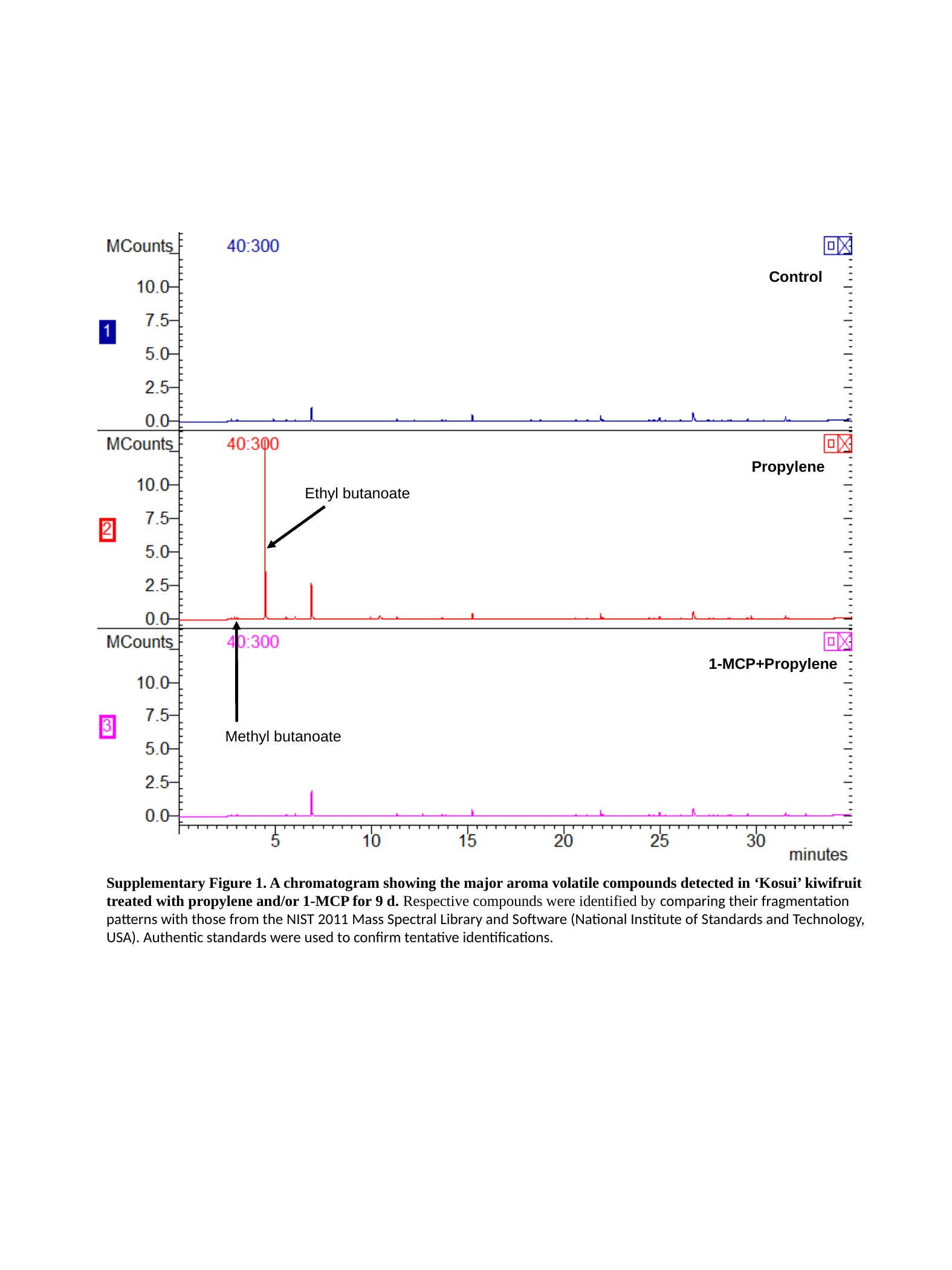

Control
Propylene
Ethyl butanoate
1-MCP+Propylene
Methyl butanoate
Supplementary Figure 1. A chromatogram showing the major aroma volatile compounds detected in ‘Kosui’ kiwifruit treated with propylene and/or 1-MCP for 9 d. Respective compounds were identified by comparing their fragmentation patterns with those from the NIST 2011 Mass Spectral Library and Software (National Institute of Standards and Technology, USA). Authentic standards were used to confirm tentative identifications.
