# Supplementary material for: Low Temperature Storage Stimulates Fruit Softening and Sugar Accumulation Without Ethylene and Aroma Volatile Production in Kiwifruit
Source: Front Plant Sci. 2019 Jul 5;10:888. doi: 10.3389/fpls.2019.00888 (PMC6625211; doi:10.3389/fpls.2019.00888)
Supplement: Supplementary file 1 [file Data_Sheet_1.ZIP › Supplementary material/Supplementary Table 1.docx]

**Supplementary Table 1. Oligonucleotide sequences of primers used for RT-qPCR analysis in this study.**

| **Name** | **Gene ID** | **Description** |  | **Sequence (5' to 3')** | **Reference** |
| --- | --- | --- | --- | --- | --- |
| *AcACS1* | Achn364251 | 1-Aminocyclopropane-1-carboxylic acid synthase | 5' to 3’ | GAAAGGCTGCGTGCAATTCTC | Asiche et al. (2018) |
|  |  |  | 3’ to 5’ | CCTGAAAATGGACTGCCCATC |  |
| *AcACO2* | Achn326461 | 1-Aminocyclopropane-1-carboxylic acid oxidase | 5' to 3’ | TCTCAGAAATCCCCGATCTCG | Asiche et al. (2018) |
|  |  |  | 3’ to 5’ | TTGGAGCCACTGAAAGCCTTC |  |
| *AcPG* | Achn051381 | Polygalacturonase | 5' to 3’ | TGGATTTGTTAGGGGTGTGC | Asiche et al. (2018) |
|  |  |  | 3’ to 5’ | CAACTTGTGTCGCTGATGAC |  |
| *AcEXP1* | Achn336951 | Expansin | 5' to 3’ | CGTGCTTCGAGCTAAAGTGC | Asiche et al. (2018) |
|  |  |  | 3’ to 5’ | CGGCGATCTTGAGGAACATG |  |
| *Acβ-AMY1* | Achn141771 | β-Amylase 1 | 5' to 3’ | CCCCACATTGATGGAATGAC | Asiche et al. (2018) |
|  |  |  | 3’ to 5’ | GTTTGTGATGCTGCCACTCG |  |
| *Acβ-AMY2* | Achn212571 | β-Amylase 2 | 5' to 3’ | CAGAGAACGCAAACTGCTCG | Asiche et al. (2018) |
|  |  |  | 3’ to 5’ | GTTCCCGGAGTCTGATCTAC |  |
| *AcINV3-1* | Achn319711 | Vacuolar invertase | 5' to 3’ | CATAGTCCTTGCCGACCATT | Nardozza et al. (2013) |
|  |  |  | 3’ to 5’ | GAGCGAAGCTCTCCACAATC |  |
| *AcAAT* | KJ626345 | Alcohol acyl transferase | 5' to 3’ | GAAATGTCTTTGCCCTTCCG | Asiche et al. (2018) |
|  |  |  | 3’ to 5’ | CCCCACCCAAAATCTATCGC |  |
| *AcNAC3* | Achn134171 | NAC transcription factor 3 | 5' to 3’ | ATTGACAAGCCGGTGCTCA | Asiche et al. (2018) |
|  |  |  | 3’ to 5’ | TGGCTTGGATTTCGCTCTG |  |
| *AcNAC5* | Achn169421 | NAC transcription factor 5 | 5' to 3’ | GAATTGCCCGAGAAAGCAGA | Asiche et al. (2018) |
|  |  |  | 3’ to 5’ | TGTCTTGATACCCTTCGGTGG |  |
| *AcMADS2* | Achn235371 | MADS-box transcription factor | 5' to 3’ | GGACAAGAACAGTCGCCAGG | Mitalo et al. (2018) |
|  |  |  | 3’ to 5’ | GTATCTGTCGCCGGTGATG |  |
| *AcACTIN* | EF063572 | Actin; housekeeping gene | 5' to 3’ | TGGAATGGAAGCTGCAGGA | Asiche et al. (2018) |
|  |  |  | 3’ to 5’ | CACCACTGAGCACAATGTTGC |  |
